# Quiz Compend

**Published:** 1898-12

**Authors:** 


Quiz Compend. Diseases of the Skin. By Jay F. Schamberg, A. B., M. D., Associate in Skin Diseases, Philadelphia Polyclinic; Dermatologist to the Union Museum Hospital, etc. Pp. 307; ninety-nine illustrations. Philadelphia: P. Blakiston's Son & Co., 1012 Walnut street.

This is a compact work for the special use of busy practitioners and students. The classifications followed is that of Duhring, which is acknowledged by all to be one of the best. Theories are excluded. One is impressed with the various points calculated to be helpful in diagnosis; especially that part which is directed to differential diagnosis, which is important to the student.

The proper treatment is suggested in a manner which allows of no misunderstanding. This is a great improvement upon average works of its kind. The purely original illustrations are of doubtful utility, as the characteristics of what they purport to represent are not properly brought out as they should be. We have no hesitation in claiming that the book as a compend, both from a scientific and practical point of view, is one of the best medical booklets yet published.

